# Identification of Stress Associated microRNAs in *Solanum lycopersicum* by High-Throughput Sequencing

**DOI:** 10.3390/genes10060475

**Published:** 2019-06-21

**Authors:** María José López-Galiano, Vicente Sentandreu, Amparo C. Martínez-Ramírez, Carolina Rausell, M. Dolores Real, Gemma Camañes, Omar Ruiz-Rivero, Oscar Crespo-Salvador, Inmaculada García-Robles

**Affiliations:** 1 Department of Genetics, University of Valencia, Burjassot, 46100 Valencia, Spain; maloga2@uv.es (M.J.L.-G.); carolina.rausell@uv.es (C.R.); maria.dolores.real@uv.es (M.D.R.); oruizrivero@gmail.com (O.R.-R.); 2 Servicios Centrales de Soporte a la Investigación Experimental (SCSIE), University of Valencia, Burjassot, 46100 Valencia, Spain; vicente.sentandreu@uv.es (V.S.); amparo.martinez@uv.es (A.C.M.-R.); 3 Plant Physiology Area, Biochemistry and Biotechnology Laboratory, Department CAMN, University Jaume I, 12071 Castellón, Spain; camanes@uji.es; 4 Department of Biochemistry and Molecular Biology, University of Valencia, IATA (CSIC), Paterna, 46980 Valencia, Spain; oscar.crespo@uv.es

**Keywords:** miRNAs, high-throughput sequencing, *Solanum lycopersicum*, biotic and abiotic stress response, differential expression, miRNA targets, hexanoic acid

## Abstract

Tomato (*Solanum lycopersicum*) is one of the most important crops around the world and also a model plant to study response to stress. High-throughput sequencing was used to analyse the microRNA (miRNA) profile of tomato plants undergoing five biotic and abiotic stress conditions (drought, heat, *P. syringae* infection, *B. cinerea* infection, and herbivore insect attack with *Leptinotarsa decemlineata* larvae) and one chemical treatment with a plant defence inducer, hexanoic acid. We identified 104 conserved miRNAs belonging to 37 families and we predicted 61 novel tomato miRNAs. Among those 165 miRNAs, 41 were stress-responsive. Reverse transcription quantitative PCR (RT-qPCR) was used to validate high-throughput expression analysis data, confirming the expression profiles of 10 out of 11 randomly selected miRNAs. Most of the differentially expressed miRNAs were stress-specific, except for *sly-miR167c-3p* upregulated in *B. cinerea* and *P. syringae* infection, *sly-newmiR26-3p* upregulated in drought and Hx treatment samples, and *sly-newmiR33-3p*, *sly-newmiR6-3p* and *sly-newmiR8-3p* differentially expressed both in biotic and abiotic stresses. From mature miRNAs sequences of the 41 stress-responsive miRNAs 279 targets were predicted. An inverse correlation between the expression profiles of 4 selected miRNAs (*sly-miR171a*, *sly-miR172c*, *sly-newmiR22-3p* and *sly-miR167c-3p*) and their target genes (*Kinesin*, *PPR*, *GRAS40*, *ABC transporter*, *GDP* and *RLP1*) was confirmed by RT-qPCR. Altogether, our analysis of miRNAs in different biotic and abiotic stress conditions highlight the interest to understand the functional role of miRNAs in tomato stress response as well as their putative targets which could help to elucidate plants molecular and physiological adaptation to stress.

## 1. Introduction

In their natural environment, plants are continuously challenged by human actions and abiotic stresses mainly associated with climate change, or biotic hazards as microbial pathogens, insects or herbivores [1,2]. Plant growth and development in response to environmental cues implicates intricate hormonal regulatory networks [3] and plant gene expression is highly regulated to cope with changes during development and adaptation to different stresses [4].

The availability of plant reference genomes and the advent of high-throughput sequencing technologies have provided new ways to understand the genetic regulation of plant processes from an omic perspective [5], including studies aimed at the comprehensive analysis of small RNAs (sRNAs) by means of profiling endogenous sRNA. sRNAs are currently classified into various groups: MicroRNAs (miRNAs), hairpin derived siRNAs (hp-siRNAs), natural antisense siRNAs (natsiRNAs), secondary siRNAs and heterochromatic siRNAs (hetsiRNAs). The large diversity of sRNA pathways in plants might be an important feature conferring phenotypic plasticity since most sRNA types play relevant roles in defense responses and in epigenetic regulation, although their relative contribution varies among different plant species [6]. In Solanaceae plants, which include more than 3000 species and are among the most economically important plants together with grasses and legumes [5], conserved regulatory cascades triggered by specific miRNAs have been characterized [7], and extensive efforts are devoted to identify miRNAs important for stress tolerance mediated by post-transcriptional stress silencing.

miRNAs are endogenous non-coding small RNA molecules, 20 to 24 nt long [8] generated from primary miRNA transcripts (pri-miRNA) that are mainly transcribed by DNA-dependent RNA-polymerase II [9,10]. In plants, pri-miRNAs are generally processed by DICER-LIKE 1 (DCL1) proteins/complexes within the nucleus with the assistance of some cofactors to produce duplexes with a complementary strand of miRNA (miRNA/miRNA*) [10]. Then, most miRNAs are loaded onto ARGONAUTE 1 (AGO1), which lies at the core of RNA-induced silencing complex (RISC) to target coding RNAs by sequence complementarity [11]. As a result, target gene expression is repressed through translational inhibition and/or RNA cleavage [9,12]. In plants, it has been shown that most target transcripts only contain one perfectly complementary site of a single miRNA located anywhere along the target mRNA instead of at the 3′-UTR as in animals [13], and base-pairing leads to degradation of the target mRNA by site-specific cleavage [8]. In contrast, when pairing to the target mRNA is imperfect translational repression occurs [14,15].

Due to their pivotal role in developmental and their trans-regulation functions, miRNAs are ideal candidates to regulate the crosstalk among hormonal signaling pathways [3] as well as gene expression networks, since miRNA main targets are transcription factors that are themselves master controllers of gene expression cascades. Besides the crucial roles of miRNAs in essential processes such as maintenance of genome integrity, signal transduction, hormone homeostasis and innate immunity, they are involved in abiotic and biotic stress responses [16]. Similar to other regulator molecules controlling pathways that cross-talk in multiple plant responses, the comparative analysis of miRNA profiles of plants undergoing different stresses constitutes an ideal strategy to obtain deep insight into the integrated regulation of stress responses.

In this work, we identified and analyzed by high-throughput sequencing miRNAs associated with stress response in tomato (*Solanum lycopersicum*), a model species for Solanaceae. Thirty-seven miRNA families were detected in tomato plant leaves undergoing six different stress conditions, and twelve miRNAs were found differentially expressed under drought (5 miRNAs), heat (5 miRNAs), CPB damage (6 miRNAs), *P. syringae* infection (15 miRNAs), *B. cinerea* infection (3 miRNAs) and Hx acid treatment (12 miRNAs). In addition, the expression fold-change of 11 stress-responsive miRNAs was validated by RT-qPCR and four of them were selected to assess the expression of their predicted targets.

## 2. Materials and Methods

### 2.1. Plant Material and Growth Conditions

Four-week-old tomato plants of *Solanum lycopersicum* Mill. cv. Ailsa Craig were grown from germinated seeds in a growth chamber, under the following conditions: 16/8 h light/night cycle, 26/18 °C day/night temperature cycle, and 60% relative humidity (RH). Seeds were irrigated twice a week with distilled water during the first week and with Hoagland solution thereafter [17].

For hexanoic acid (Hx) treatment, pots (748 cm^3^) containing 1-month-old tomato plants were either watered with 50 mL of 20 mM Hx or plain water in the corresponding mocks (non-treated plants). After 48 h, leaf tissue from 3rd and 4th leaves was harvested, frozen in liquid nitrogen, and stored at −80 °C.

For *Leptinotarsa decemlineata* (Colorado potato beetle, CPB) infestation, 15 CPB larvae of different developmental stages were placed on the 3rd and 4th leaves of thirty-day-old tomato plants. When necessary, non-cooperative larvae (molting or not eating) were removed and substituted. Leaf tissue left after 3 h of CPB feeding and that of the non-infested control plants were harvested, frozen in liquid nitrogen, and stored at −80 °C.

For drought stress experiments, tomato plants where deprived of water during one week and leaf tissue from 3rd and 4th leaves was collected, frozen in liquid nitrogen, and stored at −80 °C.

For fungi stress, *Botrytis cinerea* conidia were collected from 10- to 15-day-old Potato Dextrose Agar PDA plates supplemented with 40 mg mL^−1^ of tomato leaves and were maintained for 2 h in the dark with no shaking in Gambor’s B5 medium (Duchefa, Haarlem, The Netherlands), supplemented with 10 mM sucrose and 10 mM KH_2_PO_4_. Plants (four-week-old) were challenged by applying 5 μL droplets of 1 × 10^6^ spores mL^−1^, were maintained at 100% RH, and visible necrosis appeared 48 h after inoculation. Leaf tissue from 3rd and 4th leaves was collected 24 h post infection (hpi), frozen in liquid nitrogen, and stored at −80 °C.

For bacterial stress, *Pseudomonas syringae* pathovar tomato DC3000 was grown at 28 °C in King’s B medium (KB) [18] with Rifampicin (50 mg mL^−1^). For inoculation, *P. syringae* bacterial suspensions were adjusted to 5 × 10^5^ colony-forming units (CFU) mL^−1^ as described previously [19]. Pathogen inoculation was performed by dipping the 3rd and 4th leaves into the bacterial suspension. Leaf tissue from 3rd and 4th leaves was collected 48 hpi, frozen in liquid nitrogen, and stored at −80 °C.

For heat stress, tomato plants were grown in the above conditions during 2 weeks and the next two weeks the temperature in growth chamber was increased 5 °C (31/23 °C, light/night cycle). Leaf tissue from 3rd and 4th leaves was collected, frozen in liquid nitrogen, and stored at −80 °C.

For each stress condition, three biological replicates were generated for each treatment and their corresponding controls. A biological replicate was defined as a pool of leaf tissue from 3rd and 4th leaves from 25 plants.

### 2.2. RNA Isolation

Total RNA was isolated from leaves of tomato plants undergoing 6 different stress conditions (drought, heat, CPB damage, *P. syringae* infection, *B. cinerea* infection and Hx treatment), using RiboPure Kit (Ambion. Carlsbad, CA, USA), following the manufacturer’s protocol. To remove contaminating genomic DNA from RNA preparations, TURBO DNA-free kit (Ambion, Carlsbad, CA, USA) was used. RNA quality was assessed with the Agilent 2100 Bioanalyzer using the Nano RNA Chip Kit (Agilent, Santa Clara, CA, USA) and RIN value was obtained for each sample. Only samples having a RIN value equal or higher than 7 [20] were used for high-throughput sequencing experiments.

### 2.3. Small RNA Libraries Construction and High-Throughput Sequencing

A total of six groups of RNA samples (drought, heat, CPB damage, *P. syringae* infection, *B. cinerea* infection and Hx treatment) each with three biological replicates, and the corresponding control for each group were prepared. Thirty-six total RNA samples were used to construct sRNA libraries. sRNA fraction was enriched using the Pure Link miRNA Isolation kit (Invitrogen, Carlsbad, CA, USA). The amount and quality of miRNAs in the samples was assessed with the Agilent 2100 Bioanalyzer using the Small RNA Chip Kit (Agilent Santa Clara, CA, USA). sRNA libraries were constructed with the SOLID Total RNA-Seq Kit (Life Technologies™, Carlsbad, CA, USA). Sequencing adapters were directly ligated to the sRNA, and subsequent reverse transcription to cDNA was carried out. In this way, strand orientation is preserved and the strand a miRNA derives from can be exactly mapped. For sequencing both strands simultaneously in a single lane, barcodes were used to generate the libraries. Then, cDNA libraries were prepared and clonal amplification was performed by emulsion PCR using SOLID EZ beads System. Amplified beads were enriched and loaded in 6 lanes on the 5500xl Genetic Analyzer System (Life Technologies™). ECC (Exact Call Chemistry) module (Life Technologies™) was employed for 50 bases length sequencing.

### 2.4. Identification of Known and Novel miRNA

Raw data were preprocessed using XSQ Tools (Life Technologies™) obtaining FASTQ files. Adapter sequences were trimmed with Cutadapt software (version 1.8.3) and sequences were filtered by discarding untrimmed reads and reads of fewer than 15 nucleotides. FASTQC (v.0.11.5) was used to assess data quality (http://www.bioinformatics.babraham.ac.uk). Annotation of sRNA, known miRNAs identification and novel miRNAs prediction was conducted with the sRNAbench tool from sRNAtoolbox webserver (http://bioinfo5.ugr.es/srnatoolbox), applying the hierarchical genome mapping mode, and selecting both the tomato *S. lycopersicum* reference sequences (SL2_40) and miRBase (release 22.1, http: //www.mirbase.org/). In hierarchical mode, all reads that map to a given library are removed from the analysis and can therefore not map again. In this mode, each read can map only to one annotation group.

### 2.5. miRNA Validation by Reverse Transcription Quantitative PCR (RT-qPCR)

RNA samples used for SOLID sequencing were also employed for miRNA validation by RT-qPCR analysis. Total RNA was reverse transcribed to complementary DNA (cDNA) after polyadenylation. For each sample 1000 ng of RNA were polyadenylated in a final volume of 10 μL including 1 μL of 10x poly(A) polymerase buffer, 1 mM of ATP and 1 unit of poly(A) polymerase (New England Biolabs, Ipswich, MA, USA ) and incubated at 37 °C for 15 min and then at 65 °C for 20 min. Five hundred ng of polyadenylated RNA were used for 1st strand cDNA synthesis with the Universal primer described in Balcells et al. [21] Reverse transcription reaction was performed using PrimeScript^TM^ RT reagent Kit (Takara, Kusatsu, Japan) in a final volume of 10 μL, including 2 μL of 5X PrimeScript^TM^ Buffer, 0.5 μL of PrimeScript^TM^ RT Enzyme Mix I and 1 μM of Universal_RT-primer and it was incubated at 37 °C for 15 min followed by enzyme inactivation at 85 °C for 5 s. The sequence of the RT-primer (Integrated DNA Technologies, Coralville, IA, USA) was 5′-CAGGTCCAGTTTTTTTTTTTTTTTVN-3′, where V is A, C and G and N is A, C, G and T.

RT-qPCR was carried out using 10 ng of cDNA on a StepOnePlus Real-Time PCR system (Life Technologies™, Carlsbad, CA, USA) thermocycler, following the manufacturer’s instructions, using Power SYBR Green PCR Master Mix (Applied Biosystems, Foster City, CA, USA). The cycling parameters were: initial polymerase activation step at 95 °C for 10 min, 40 cycles of denaturation at 95 °C for 15 s, annealing and elongation at 60 °C for 1 min. Specific forward and reverse primers were designed according to Balcells et al. [21]. The list of RT-qPCR primers for stress miRNA amplification can be found in Additional file1, Table S1.

Three biological replicates (with 3 technical replicates each) were analyzed and *U6* snRNA gene (GenBank: X51447.1) was used to normalize miRNA expression.

LinRegPCR software [22] was employed for the analysis of RT-qPCR experiments and data were analyzed using Student’s *t*-test for statistically significant differences (*p* < 0.05).

### 2.6. miRNA Target Prediction and Function Analysis

Target genes of stress-responsive miRNAs in tomato were predicted using the psRNATarget online tool (http://plantgrn.noble.org/psRNATarget/) and miRNAconsTarget tool from sRNAtoolbox (http://bioinfo5.ugr.es/srnatoolbox). Default parameters for target prediction were used.

Gene ontology enrichment analysis of the identified target transcripts was executed with the online tool AgriGO (GO Analysis Toolkit and Database for Agricultural Community (http://bioinfo.cau.edu.cn/agriGO/). Three important components such as biological process, cellular component, and molecular function associated with each GO term were inferred.

### 2.7. miRNA Target Validation by RT-qPCR

RNA samples used for SOLID sequencing were also employed for miRNA target validation by RT-qPCR analysis. Total RNA was reverse transcribed to complementary DNA (cDNA) with RetroScript Kit (Ambion, Carlsbad, CA, USA) following manufacturer instructions with 50 ng/μL oligo (dT)15 (Promega, Madison, WI, USA) and 2.5 μM random hexamers (Applied Biosystems) and amplification was carried out on a StepOnePlus Real-Time PCR system (Applied Biosystems) thermocycler, using Power SYBR Green PCR Master Mix (Applied Biosystems, Foster City, CA, USA). The cycling parameters were: initial polymerase activation step at 95 °C for 10 min, 40 cycles of denaturation at 95 °C for 15 s, annealing and elongation at 60 °C for 1 min. For each sample, three biological replicates (with 3 technical replicates each) were analyzed and *RPS18* gene (ribosomal protein S18, Gene ID: 107882131) was used to normalize gene expression.

LinRegPCR software [22] was employed for the analysis of RT-qPCR experiments and data were analyzed by Student’s *t*-test for statistically significant differences (*p* < 0.05).

The list of RT-qPCR primers for miRNA target genes amplification can be found in Additional file 1, Table S1.

### 2.8. miRNA Differential Expression Analysis

Differential expression analysis of the mature miRNAs was performed with DESeq package implemented in the sRNAde tool from sRNAtoolbox server (http://bioinfo5.ugr.es/srnatoolbox). miRNAs exhibiting padj value < 0.1 (*p*-value adjusted for multiple testing using Benjamini–Hochberg method [23]) were further characterized.

The sequence data were deposited in the NCBI Short Read Archive (SRA) with the accession number: SRP113520, BioProject: PRJNA395638 and BioSamples: SAMN07411887 to SAMN07411922.

## 3. Results

### 3.1. Overview of Small RNAs Distribution in Tomato Plants under Six Different Stress Conditions

A total of 36 sRNA libraries from tomato plants comprising 6 stress conditions (drought, heat, CPB damage, *P. syringae* or *B. cinerea* infection, and Hx treatment) were generated and sequenced using the SOLID technology (Life Technologies™).

**Figure 1:**
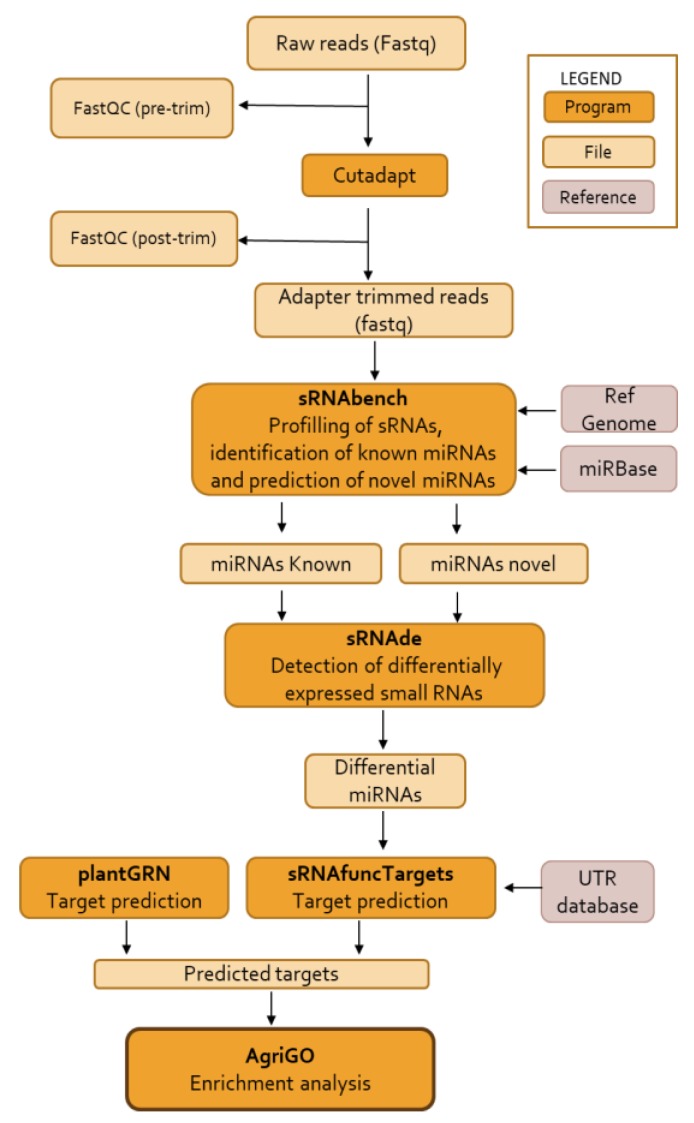
Pipeline workflow of *S. lycopersicum* miRNA search and target prediction. Pre-trim: pre-trimming, post –trim: post-trimming, Ref genome: reference genome, miRBase: micro RNAs database, miRNAs: micro RNAs, sRNAs: small RNAs, UTR database: untranslated region database.

Figure 1 shows an overview of the workflow followed to process raw data and predict miRNAs and their targets. First, to obtain high quality data sets, adaptors and low-quantity reads were removed and we obtained 0.23 to 9.5 million clean reads between 15 and 47 nt in length from each of the 36 libraries. For each library, details of raw reads and clean reads are shown in Table 1.

**Figure 2:**
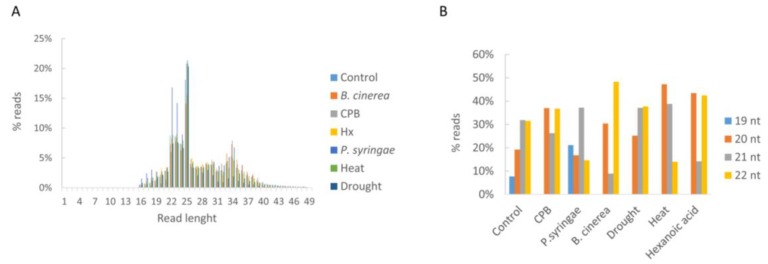
Size distribution of total reads in the thirty-six libraries used in this work. (**A**) Read length distribution for each stress condition. (**B**) Distribution of 19 to 22 nt miRNA assigned reads. Control reads correspond to an average of all control plants across treatments.

The size distribution of the sRNAs is shown in Figure 2A. Reads of 19 to 24 nt long accounted for over 40% of the total reads, among which 24 nt long reads were the most abundant in all libraries (14–21%).

**Figure 3:**
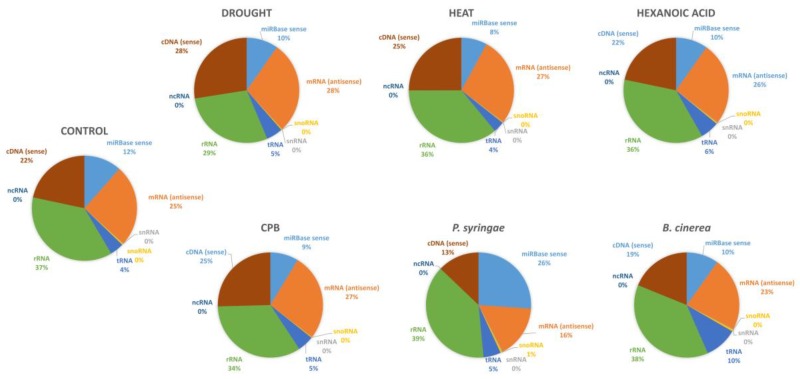
An overview of the frequency of the different small RNAs (sRNA) species present in libraries corresponding to the stress groups. snoRNA, small nucleolar RNA; miRNA, microRNA; tRNA, transfer RNA; rRNA, ribosomal RNA; snRNA, small nuclear RNA; ncRNA, non-coding RNA; complementary DNA cDNA (sense). Control reads correspond to an average of all control plants across treatments.

To sort out the sRNA sequenced reads into categories and identify miRNA sequences in the 36 libraries, reads were mapped to specific databases (miRBase, cDNA, tRNA, and rRNA databases). The sequenced sRNA reads that mapped to the miRBase database were mainly 20–22 nt long for all libraries (Figure 2B). sRNA reads mapped to the miRBase database were more abundant in *P. syringae* libraries (26%) compared to the rest of libraries that ranged from 8% to 12% (Figure 3). A similar frequency distribution of different sRNA species was obtained after mapping to other databases (Figure 3). Reads that mapped to cDNA (sense) database ranged from 13% to 28%, reads mapped to tRNA database were within 4–10%, and 29% to 38% reads mapped to the rRNA database.

Table 1 summarizes the total number of sequences that matched *S. lycopersicum* genome.

### 3.2. Identification of Known miRNAs

Conserved miRNAs in tomato were identified by mapping sRNA sequences obtained from each library to the miRNAs database (miRBase 22.1, released in October 2018) selecting *S. lycopersicum*, *S. tuberosum*, *A. thaliana*, and *N. tabacum* known miRNAs. Homology search was performed and miRNAs with low expression levels (less than 2 reads) were removed. An average of 85 known miRNAs were identified in *P. syringae* and *B. cinerea* infected samples, and drought or heat stress samples but only 64 and 28 known miRNAs were detected in CPB infested samples and Hx treated samples, respectively (Table 2). A total of 100 known miRNAs belonging to 37 miRNA families were identified in the 36 libraries being *sly-miR482e-3p*, *sly-miR167a*, and *sly-miR159* those having the largest number of reads in each library (Additional file 2, Table S2).

Details of known miRNAs of each library are listed in Additional file 3, Table S3. Among identified miRNAs, *sly-miR482* and *sly-miR171* families contained the highest number of members (7 and 6, respectively).

### 3.3. Identification of Novel miRNAs

A total of 62 novel miRNAs were identified from the 36 sRNA sequenced libraries using sRNAbench tool from sRNAtoolbox webserver (Table 3). Detailed information of predicted novel miRNAs for each library is presented in Additional file 4, Table S4.

All novel predicted miRNAs were selected with the default parameters of sRNAbench, including the presence of reads from 3p-arm and 5p-arm mature sequences, the duplex formation among those sequences and the presence at the tomato genome of a premiR sequence that could be folded into a hairpin-like structure, similar to those of other miRNAs. Only miRNAs that fulfill those criteria were consider as novel tomato miRNAs. The precursor sequence of these new miRNA candidates varied from 54 to 200 nt in length and the MFE of precursor hairpins ranged from −7.20 to −152.7 kcal/mol.

**Figure 4:**
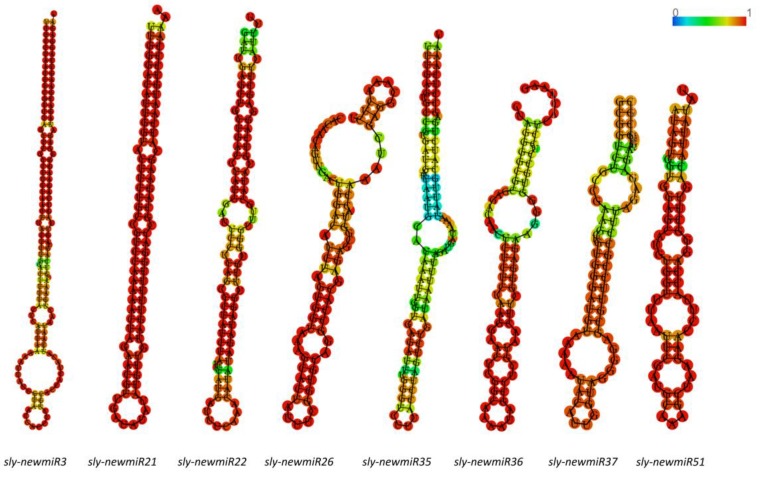
Precursor hairpin structures of eight representative novel *S. lycopersicum* miRNAs. Mature miRNA sequences are shown. Color gradient indicates base-pair probabilities.

Sequence and characteristics of eight representative novel miRNAs predicted at RNAfold web server (http://rna.tbi.univie.ac.at//cgi-bin/RNAWebSuite/RNAfold.cgi) are shown in Table 4, and their precursor sequences, as well as the stem-loop hairpin secondary structure are depicted in Figure 4. 

### 3.4. Stress-Responsive microRNAs

To identify stress-responsive miRNAs, normalized expression profiles of known and novel miRNAs in stress samples were compared to their corresponding control samples using DESeq package implemented in the sRNAde tool from sRNAtoolbox. mirRNAs showing a fold change of 2 and a *p*(adj)-value < 0.1 were considered differentially expressed (Table 5, Additional file 5: Table S5).

**Figure 5:**
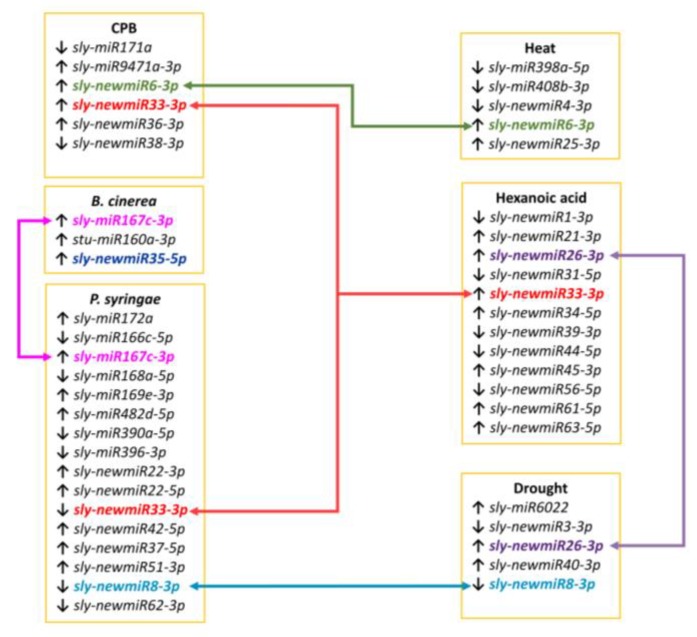
Schematic representation of differentially expressed miRNAs in six stress conditions. Connector lines link miRNAs detected in two or more stress conditions.

A total of 41 miRNAs were found differentially expressed and predominantly upregulated in stress samples comparing to controls. *P. syringae* and *B. cinerea* samples displayed the highest (13) and lowest (3) number of stress-responsive miRNAs, respectively. Thirty-six out of the 41 differentially expressed miRNAs were only identified in one stress condition whereas the remaining 5 responsive miRNAs were not stress specific and were identified in two biotic stresses (*sly-miR167c-3p* in *B. cinerea* and *P. syringae* samples), or two abiotic stresses (*sly-newmiR26-3p* in drought and Hx treatment samples), or in both biotic and abiotic stresses (*sly-newmiR33-3p*, *sly-newmiR6-3p* and *sly-newmiR8-3p*) (Figure 5). Intriguingly, all miRNAs differentially expressed detected in samples treated with the plant defense inducer Hx were novel miRNAs.

### 3.5. miRNA Validation

**Figure 6:**
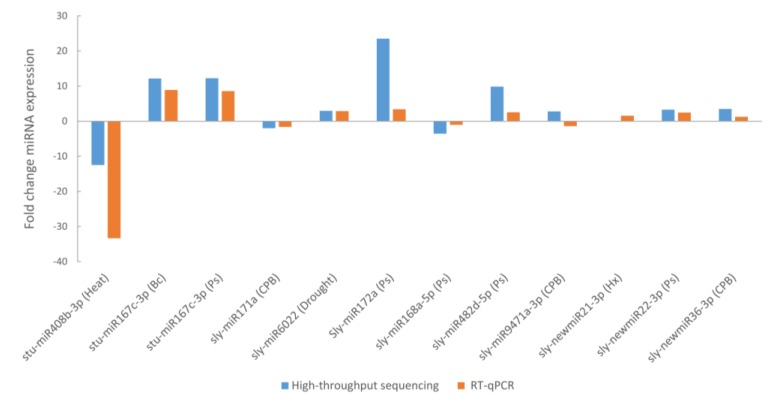
Expression levels of 11 selected stress-responsive miRNAs determined by high-throughput sequencing and RT-qPCR analysis. Selected miRNAs corresponded to 7 known stress-specific miRNA, 1 known miRNA differentially expressed in two stress conditions (*sly-miR167c-3p* in *B. cinerea* (Bc) and *P. syringae* (Ps) infections) and 3 novel stress-specific miRNAs.

Eleven out of the 41 miRNAs that were found differentially expressed were selected for validation of the high-throughput sequencing analysis by qRT-PCR, eight known miRNAs (*sly-miR408b-3p*, *sly-miR167c-3p*, *sly-miR171a*, *sly-miR6022*, *sly-miR172c*, *sly-miR168a-5p*, *sly-miR482d-5p* and *sly-miR9471a-3p*) and 3 novel miRNAs (*sly-newmiR21-3p*, *sly-newmiR22-3p* and *sly-newmiR36-3p*) (Additional file 6, Table S6). All miRNAs, except *sly-miR9471a-3p* showed the same tendency in sequencing and RT-qPCR (Figure 6). *sly-miR167c-3p* was differentially expressed in two different stresses and it was correctly validated in both conditions. Therefore, 91% of selected miRNAs were validated by RT-qPCR.

### 3.6. Stress-Responsive miRNAs: Targets and Functional Analysis

The mature sequences of the 41 differentially expressed miRNAs identified were used to search for their targets in tomato genome with the online tools psRNATarget (http://plantgrn.noble.org/v1_psRNATarget) and miRNAconsTarget from sRNAtoolbox (http://bioinfo5.ugr.es/srnatoolbox), by matching the miRNAs to *S. lycopersicum* reference genome sequence (SL2_40). A total of 87 target genes for the known miRNAs and 94 targets for the novel miRNAs were predicted (Table 6). Over 82% of the target genes identified (146) were predicted to be negatively regulated by miRNAs in a miRNA cleavage manner, while the rest (30) might be translationally repressed. Information on the target genes (including target ID and functional annotation) is shown in Additional file 7, Table S7.

The amount of targets predicted varied from one (in the case of *sly-miR167c-3p* and *sly-miR9471a-3p*) to 28 (for the novel miRNA *sly-newmiR3-5p*). A number of the target mRNAs have been described to be involved in metabolism, growth and response to abiotic and biotic stress. In addition, some of the transcripts could be potentially regulated by several miRNAs belonging to different families (Additional file 7, Table S7).

**Figure 7:**
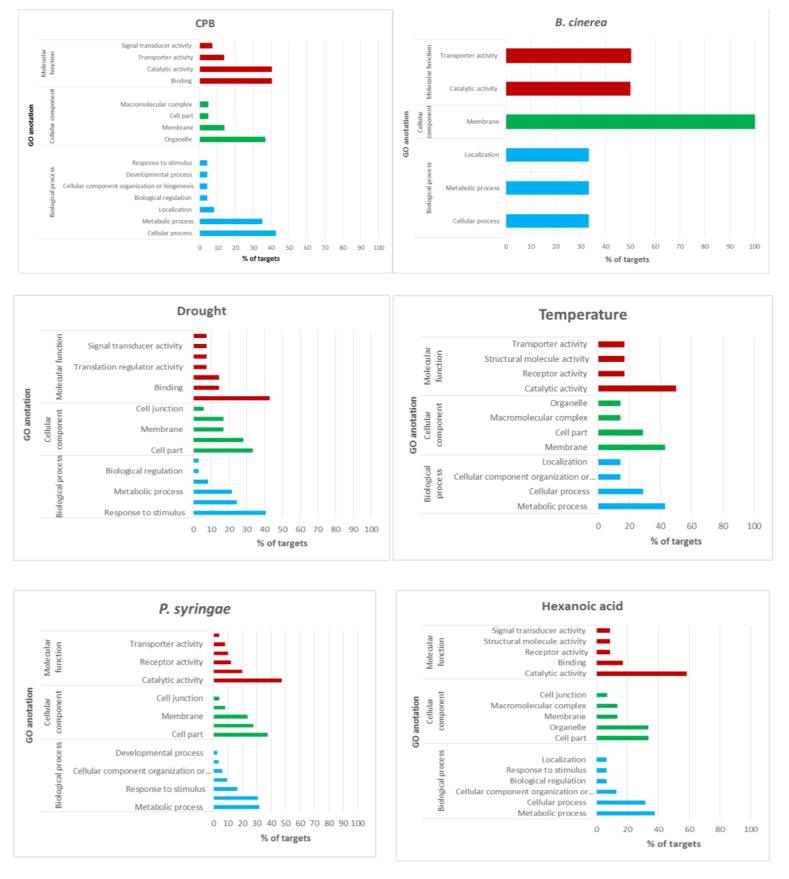
GO analysis of target transcripts regulated by stress-responsive miRNAs.

Gene ontology analysis of the miRNAs target transcripts using AgriGO allowed us to identify enriched gene ontology terms significant at 1% FDR corresponding to binding and catalytic activity in most stress conditions (Figure 7, Additional file 8: Table S8).

### 3.7. miRNA Target Validation

To verify the expression of the miRNAs target genes predicted, primers were designed to perform RT-qPCR of the following tomato genes: *Kinesin*, *PPR*, *GRAS40*, *ABC transporter*, *GDP*, *RLP1*, targeted by *sly-miR167c-3p*, *sly-miR172c*, *sly-miR171a-3p* and *sly-newmir22-3p* (Table 7).

**Figure 8:**
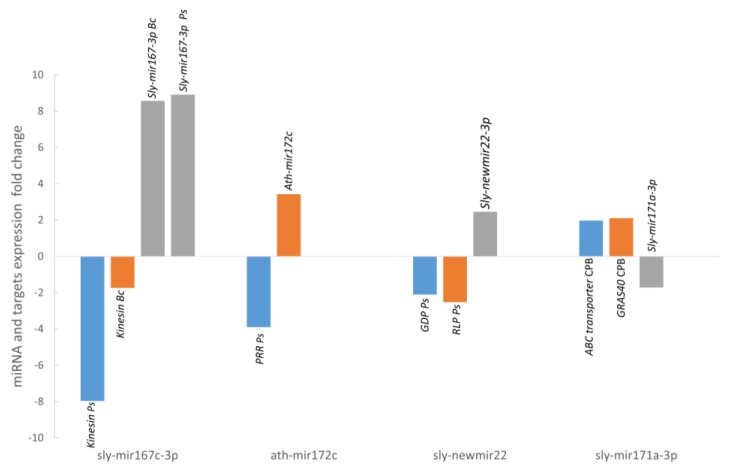
Expression levels of 4 selected miRNAs and their targets determined by RT-qPCR. Selected miRNAs are *sly-miR167c-3p* and its target, *Kinesin-like*, RT-qPCR analysis was performed in samples of *B. cinerea* stress and *P. syringae* stress. *ath-miR172c* and its target *PPR* in *P. syringae* stress. *sly-newmiR22-3p* and its targets GPD and RPL in *P. syringae* stress and *sly-newmiR171a-3p* and its targets, *ABC transporter* and *GRAS40* in CPB stress.

An inverse expression pattern was observed among the miRNAs and their target genes in all cases (Figure 8). *sly-mir167-3p*, *sly-mir172c* and *sly-newmir22-3p* were upregulated in *P. syringae* and *B. cinerea* infected samples and accordingly genes encoding *Kinesin-like*, *PPR*, *GDP*, and *RLP1* were downregulated, while *sly-mir171a-3p* was downregulated and its predicted targets *GRAS40* and *ABC transporter* were upregulated (Figure 8).

## 4. Discussion

Tomato is one of the most cultivated crops around the world [24] with an annual production of around 164 million tons [25]. Traditionally, tomato has been a research model for fruit development and since the completion of its genome sequence in 2012 [26], it offers an excellent system to study gene regulation in relation to plant stress response. Abiotic stresses, like drought, heat, cold, salinity, and biotic stresses like bacteria or fungi infection or herbivores attack dramatically affect the yield and quality of crops. Recently, the important role of sRNAs as a versatile regulation mechanism of the plant response to stress has been evidenced. In this work high-throughput sequencing of sRNAs with SOLiD technology has been used to obtain the miRNA profile of tomato plants undergoing 5 different stress conditions (drought, heat, *P. syringae* infection, *B. cinerea* infection, and herbivore insect attack with CPB larvae) or chemical treatment with the plant defense inducer Hx. We have detected 104 known miRNAs belonging to 37 families, and 62 novel tomato miRNAs. For the identification of known miRNAs, strict parameters in sRNAbench tool of the sRNAtoolbox were used and no mismatches or gaps allowed. For novel miRNAs predictions, strict default parameters were also used, and only were selected as putative novel miRNAs those having reads for both the 5p-arm and 3p-arm mature miRNAs sequences able to form a duplex, and with a pre-miR sequence mapped in the tomato genome. Read counts differed notably among conserved and novel miRNAs, with the latter displaying lower expression. A few conserved miRNA families such as *miR482*, *miR167*, and *miR159* were the most abundantly expressed (more than 27,000 RPM), accounting for 36%, 20% and 15% of all the conserved miRNA reads, respectively. When comparing miRNAs expression patterns among stress samples and controls 40 out of the 165 miRNAs identified were found stress-responsive, which might potentially be implicated in the regulation of stress response in tomato. Supporting the reliability of the miRNA detection by deep sequencing, fold change of eleven random selected differential miRNAs was consistent with the analysis by RT-qPCR (91% miRNAs validated). In all stress conditions, most stress-responsive miRNAs were upregulated (55% in the total 41 stress-responsive miRNAs detected). None of these 41 miRNAs was differentially expressed in all stress conditions, whereas *sly-miR167c-3p* expression was detected in *B. cinerea* and *P. syringae* infection, *sly-newmiR26-3p* in drought and Hx treatment samples, and *sly-newmiR33-3p*, *sly-newmiR6-3p*, and *sly-newmiR8-3p* both in biotic and abiotic stresses. Therefore, it seems that miRNAs expression during stress is dependent on the specific stress to which the plant is subjected.

Tomato stress-responsive miRNAs have been recently identified by high-throughput sequencing in specific stress conditions, such as elevated temperature [27], drought [28,29], and *B. cinerea* infection [30]. However, there has been no report addressing the global parallel identification of miRNAs in different biotic and abiotic stress conditions to understand the functional role of miRNAs in tomato stress response.

### 4.1. Heat Stress-Responsive miRNAs

In response to heat stress we found two miRNAs highly conserved in plants (*sly-miR398a-5p* and *sly-miR408b-3p*) that were both down-regulated, and three novel miRNAs. By tomato deep sequencing, Zhou et al. [27] reported repression of *sly-miR408b-3p* expression at moderately and acutely elevated temperature (−1.02 and −1.70 Log_2_ fold change, respectively) whereas *sly-miR398a-5p* was only significantly down-regulated at acutely elevated temperatures. In *Capsicum annuum miR408* and *miR398* families were also down-regulated in response to high temperature [31]. In *Arabidopsis*, *miR408* and *miR398* and their target genes, including Cu/Zn superoxide dismutases (*CSD1* and *CSD2*) and the copper chaperone *CCS1* [32,33] have been extensively studied in relation to diverse abiotic stresses (cold, salinity, drought, oxidative and osmotic stress) demonstrating a negative correlation between these miRNAs and *CSD1*, *CSD2* and *CCS1* target mRNAs.

### 4.2. Drougth Stress-Responsive miRNAs

In the current study four novel miRNAs and one conserved miRNA (*sly-miR6022*) were found drought-responsive. Candar-Cakir et al. [28] detected *sly-miR6022* among the miR families represented with the top read abundance in two different drought-responsive tomato cultivars (sensitive vs. tolerant), being up-regulated in the drought tolerant genotype in response to water deprivation. When profiling drought-responsive microRNAs in other sensitive and tolerant tomato lines, Liu et al. [29] despite identifying *sly-miR6022* did not detect statistically significant differential expression between stress samples and controls. This suggests that different plant species or even cultivars may behave distinctively in plant stress responses regulated by miRNA. It has been proposed that the receptor like protein (RLP) containing leucine-rich repeat (LRR) is targeted by *miR6022* not only in tomato plants undergoing abiotic stresses [27] but also in response to plant pathogens [34].

### 4.3. B. cinerea Stress-Responsive miRNAs

It has been reported that plants miRNAs such as *miR160*, *miR167* and *miR393* are involved in disease resistance by coordinating plant hormone regulatory networks [35]. These three miRNAs were identified in a tomato deep sequencing analysis upon *B. cinerea* infection, but only *miR160* was found responsive to *B. cinerea* targeting auxin response factors (ARF) [30]. In our study, following *B. cinerea* infection expression of a novel *sly-newmiR35-5p* was up-regulated as well as that of *sly-miR167c-3p* and *sly-miR160a-3p*, corroborating their involvement in the response to *B. cinerea* infection in tomato leaves.

### 4.4. P. syringae Stress-Responsive miRNAs

*Arabidopsis* plants challenged with *P. syringae* showed similar expression profile of most of those highly and moderately conserved miRNAs detected in our study [36]. Among others, *miR396*, *miR390* and *miR166* families were down-regulated, and *miR167*, *miR169*, and *miR172* families were upregulated, while *miR168* and *miR482* were not detected in *Arabidopsis* [36].

Auxin is considered a pattern-forming phytohormone responsible of plant growth and major developmental processes, many of which are modulated by the auxin response transcription factor (ARF) family [37] targeted by *miR167* and *miR390*. While *ARF6* and *ARF8* are targets of *miR167*, expression of *ARF2*, *ARF3* and *ARF4* is regulated by *miRNA390* through TAS3-derived ta-siRNAs (trans-acting short-interfering RNAs, a class endogenous secondary siRNAs produced through the action of RNA-dependent-RNA-polymerase-6 upon microRNA-mediated cleavage of non-coding TAS RNAs) in *Arabidopsis* [38,39,40]. *sly-miR167* not only was found up-regulated after bacterial challenging with *P. syringae* in tomato (present work) and *Arabidopsis* [41] but also in response to infection with the fungal pathogen *Fusarium oxysporum* in tomato leaves [42]. In the case of *miR390*, it does not target a protein-coding mRNA, but rather triggers the production of tasiRNAs from the TAS3 locus, which in turn causes degradation of the ARF3 and ARF4 mRNAs in a miRNA-like fashion [43,44].

*miR167* and *miR390* have been also described to be responsive to ABA [45,46], a phytohormone that has been involved in the early infection stage of antibacterial defense [47]. After *P. syringae* infection we have found *miR167* and *miR390* differentially expressed, as well as *miR169*, also implicated in ABA functions [48,49].

miRNA families that target genes involved in auxin signaling and ABA response might shape a regulatory network for the molecular adaptation of tomato plants in response to *P. syringae* infection.

*sly-miR168* expression was up-regulated in tomato plants undergoing *P. syringae* infection. The *miR482/2118* superfamily is unusually diverse (at least 31 isoforms), and variable both in sequence (22 nucleotides rather than 21 nucleotides long) and in expression level. The abundance of *miR482* members varies greatly among species and families of plants, having the Solanum genera remarkably high levels of *miR482*. All variants of this superfamily target the mRNA sequences of genes coding for disease resistance proteins (Resistance-like genes) with nucleotide binding site (NBS) and leucine-rich repeat (LRR) motifs [7] for direct degradation as well as by generating secondary small interfering RNAs (siRNAs) in *P. syringae* infected *Nicotiana benthamiana* plants [50], and also in the *Arabidopsis* 22-nt *miR472* related to *miR482* [51]. In contrast, *sly-miR482d-5p* was described to target a PPO encoding gene in potato plants [52] and *sly-miR482d-5p* targets predicted in the present study were not related to defense functions.

In tomato plants challenged with *P. syringae*, *sly-miR168* expression was found reduced. It has been described that *miR168*, by regulating ARGONAUTE 1 (AGO1) homeostasis, exerts a regulatory feedback control over other miRNAs biogenesis [53]. Thus, *miR168* functioning as an initial regulator modulates the levels of miRNAs coordinating the cross talk of stress response pathways and development programs.

miRNA regulated transcription factors are major nodes coordinating plant growth and differentiation related processes, stress responses, and signaling pathways crosstalk. It is well known that *miR396* targets plant GROWTH-REGULATING FACTORS (GRFs) implicated in the regulation of leaf growth, *miR172* binds to the 3′-end of APETHALA 2 (AP2) domain transcription factors, which are involved in flowering time control, and *miR166* target HOMEODOMAIN LEUCINE ZIPPER (*HD-ZIP*) transcription factors family genes that regulate plant shoot apical meristems development [54]. Moreover, members of the NF-Y family of transcription factors that play crucial roles in development and in response to adverse environmental conditions are targeted by *miR169* [48]. Tomato plants challenged with *P. syringae* showed altered profiles of all those miRNAs indicating that physiological adaptation to pathogen attack requires an integrated expression of genes responsible of immune defense and growth.

### 4.5. CPB Stress-Responsive miRNAs

Very few studies addressed miRNA identification by high-throughput sequencing in plants attacked by insects. We have detected two conserved miRNAs (*sly-miR171a* and *sly-miR9471a-3p*) and four novel miRNAs differentially expressed in CPB infested tomato plants. *miR171* family was significantly increased in wounded *Nicotiana attenuata* leaves treated with *Manduca sexta* oral secretions whereas *miR171* potential targets GRAS proteins were down-regulated [55]. Among other miRNAs, *miR171* was considered as a JA-independent miRNA [55]. In contrast, Gao et al. [56] profiling miRNAs under wound treatment in *Aquilaria sinensis* found consistently lower expression levels of *miR171* family members in wounded stems compared to healthy stems. Similarly, in our study, *sly-miR171a* was repressed in tomato plants damaged by CPB. Regarding *sly-miR9471a-3p*, this miRNA was among the most abundant miRNAs families detected in *Alternaria alternata* infected tomato plants, although no differential expression was observed comparing to control plants [57]. 

### 4.6. miRNAs Expressed in Response to the Priming Agent Hx

As a result of exposure to stress, plants often become more resistant to future exposure through a memory acquisition process named priming, which can also be mediated by natural compounds like Hx [58]. Plant defense priming allows plants to respond to biotic and abiotic stress better than unprimed plants avoiding the fitness costs associated with permanent full defense activation [59]. No differentially expressed conserved miRNAs following Hx treatment were identified and twelve novel Hx responsive miRNAs were found.

Little is known about the molecular mechanisms underlying priming process, but sustained impaired levels of signaling molecules after the initial stress that boost extensive transcriptional reprogramming of defense genes upon further challenge, as well as histone acetylation and DNA methylation epigenetic modifications have been proposed as critical regulators of defense priming [60,61,62]. A role for miRNAs in stress memory has been recently described based on their specific functions as translational inhibitors. Induction of isoforms of the microRNA *miR156* has been reported following heat stress, and repression of their target genes was needed for the maintained enhanced expression of memory genes and for physiological heat stress memory [63]. In addition, Soto-Suárez et al. [64] provided evidence that miRNAs might be sustaining defense priming by demonstrating that reduced *miR396* levels and up-regulation of its target genes sensitized plants to mount more robust defense responses during pathogen infection even when in the absence of pathogen challenge, the transcriptome and development of modified plants with diminished *miR396* activity could not be distinguished from those in wild-type plants. 

### 4.7. miRNAs Diferentially Expressed across Stresses

To the best of our knowledge, there is no research work on global expression profiling of tomato miRNAs in response to *P. syringae* infection. We have detected eight conserved differentially expressed miRNAs one of them also responsive to *B. cinerea* infection (*sly-miR167c-3p*), and seven novel miRNAs two of which were responsive to other stresses as well (*sly-newmiR8-3p* also down-regulated in response to drought, and *sly-newmiR33-3p* responsive to CPB infestation or Hx treatment) (Figure 5). Among the eight known *P. syringae*-responsive miRNAs identified, five miRNAs (*ath-miR172c*, *sly-miR166c-5p*, *sly-miR168a-5p*, *sly-miR396-3p* and *sly-miR390a-5p*) belong to families highly conserved in plants, two are moderately conserved (*sly-miR167c-3p* and *sly-miR169e-3p*), and *sly-miR482d-5p* is a non-conserved plant miRNA. Highly and moderately conserved miRNA families have high expression levels and play relevant functions in plant development regulating gene expression of multiple targets in numerous plant species [13]. Our analysis revealed several miRNA families that target genes involved in a number of essential pathways mainly regulating auxin signaling (*miR167* and *miR390*), development and reprograming processes (*miR396*, *miR172*, *miR169* and *miR166*), and stress defense (*miR167*, *miR169* and *miR390*), especially ABA response.

In our work, we analyzed the miRNA profile of tomato plants treated with the natural defense priming inducer Hx that protects tomato against *B. cinerea* and *P. syringae* with high efficiency [65]. Interestingly, although no differentially expressed conserved miRNAs following Hx treatment were detected, two of the twelve novel Hx responsive miRNAs found (*sly-newmiR33-3p* and *sly-newmiR26-3p*) showed varied expression in tomato plants subjected to other stresses (CPB infestation and *P. syringae* infection, or drought, respectively, Figure 5). These results suggest that Hx treatment might exert its priming effect by triggering persistent expression of a complex network of miRNAs (not all of them necessarily involved in known stress signaling cascades) that upon exposure to a subsequent stress regulate an enhanced, more efficient or more rapid response. Further research will be needed to uncover the role of each Hx responsive novel miRNAs and their target genes, and decipher how they contribute to Hx defense priming.

### 4.8. Validation of Stress-Responsive miRNAs and Their Targets

A total of 181 targets genes were predicted (87 for conserved miRNAs and 94 for novel miRNAs) using the mature sequences of the 41 differentially expressed miRNAs. Most of the targets (82%) were predicted to be negatively regulated in a miRNA cleavage manner, therefore an opposite expression profile is expected for those miRNA-target pairs. We have selected 5 of these targets for validation with RT-qPCR focusing on *P. syringae* infection and CPB larvae infestation, two stress conditions for which there is a lack of information about their miRNA expression profile. We have selected targets for miRNAs that were validated with deep sequencing and RT-qPCR and that were either stress specific conserved miRNAs, *sly-miR171a* for CPB (*GRAS* and *ABC transporter*) and *sly-miR172c* for (PPR protein), stress specific novel miRNA as *sly-newmiR22-3p* for *P. syringae* infection (*GDPDL3* and *RLP1*) or multi-stress miRNA, as *sly-miR167c-3p* for *P. syringae* and *B. cinerea* infection (*Kinesin*).

*sly-miR172c* expression was upregulated in tomato plants after infection with *P. syringae* (3.89-fold). A pentatricopeptide repeat-containing protein (PPR) that belongs to the Tandem Repeats protein group is one of targets predicted for this miRNA. In *Arabidopsis*, PPR proteins is one of the largest protein families with at least 466 genes in its genome [66,67]. Many repeat protein in plants, as PPR, LRR or WD40 have a role in primary metabolism but also act as regulators in plant secondary metabolism including abiotic and biotic stress response [68,69]. In *Solanum tuberosum*, *PPR* expression was downregulated upon *Ralstonia solanacearum* infection [70] and in *Arabidopsis*, the absence of this protein led to an increase in the sensibility to a necrotrophic fungal pathogen and hypersensitivity to abiotic stresses such as salinity, glucose, and ABA [71]. In this work, *PPR* expression was downregulated in tomato leaves after *P. syringae* infection (3.42-fold).

*sly-miR67c-3p* expression was upregulated upon *P. syringae* and *B. cinerea* infection (8.91-fold, 8.57-fold, respectively) and there is only one target predicted for this miRNA, a Kinesin light chain-like protein. Kinesin is a motor protein that moves along the microtubules of the cytoskeleton. The actin cytoskeleton in plants has been proposed to be associated to plant cell shape, plant development, and stress response [72]. Both biotic and abiotic stresses might be affected by the continuous activity and reorganization of the host actin cytoskeleton [72,73]. In *Arabidopsis*, Shimono et al. [74] showed that kinesin mutant plants had less symptoms after *P. syringae* infection and they speculate with the possibility of and integrative function for kinesin, supporting cellular traffic during pathogen invasion, and immune signaling. In this work, we have found a reduction of the kinesin gene expression after *P. syringae* infection (−7.96-fold) and *B. cinerea* infection (−1.74-fold).

The expression of *sly-miR171a* miRNA was reduced in tomato plants (1.71-fold) after CPB larvae attack. Among the five predicted targets for this miRNA, three of them are members of the GRAS family of transcription factors (subfamily HAM): GRAS8, GRAS24 and GRAS40. In tomato, Huang et al. [75] by 5’-RACE analysis demonstrated the regulation of GRAS24 and GRAS40 by *sly-miR171* and in *Arabidopsis*, it had been previously reported that their closest homologous (AtSCL6, 22, 27) were post-transcriptionally regulated by ath-*miR171* [76,77]. Huang et al., [78] established the implication of GRAS24 in the regulation of gibberellin and auxin homeostasis. Maryose et al. [79] showed that transcript levels of eight tomato GRAS genes increased in response to mechanical stress. Also, a GRAS homologue in *Nicotiana attenuate* and *Solanum nigra* has been found to be induced upon *M. sexta* attack [80]. In this work we have analyzed by RT-qPCR the expression of the three GRAS genes predicted as targets of *sly-miR171a*. *GRAS8* and *GRAS24* gene expression was not affected after CPB larvae attack (data not shown) but *GRAS40* expression was increased by a 2-fold, which could account for its participation in tomato response to herbivore attack.

The other putative target of *sly-miR171a* is a multidrug resistance protein of the ABC transporter family, which use the energy released by ATP hydrolysis to drive the exchange of compounds across biological membranes even against electrochemical gradients [81]. Oral secretions of herbivore chewing insects contains compounds that can be recognized by plants triggering a defense response [82,83] in which plants accumulate secondary metabolites and inhibitory proteins to stop pathogen invasion and insect attack. There are evidences indicating that ABC transporters mediate the secretion of plant defense compounds into both the rhizosphere and the apoplast, or onto the plant surface [84]. In *Nicotiana tabacum*, Bienert et al. [85] showed that the ABCG5/PDR5 transporter expression in leaves was very low but it was induced after wounding by the herbivore *M. sexta*. In this work, the tomato ABC transporter augmented its expression (1.98-fold) in leaves following CPB larvae attack, which would be in agreement with an implication of the pair *sly-miR171a*–ABC transporter in tomato response to herbivore attack.

*sly-newmiR22-3p* expression was found increased after *P. syringae* infection (2.46-fold). Two of its predicted targets were a Receptor-like protein kinase (RLP1), which has a LRR motif, and a Glycerophosphoryl diester phosphodiesterase family protein (GDPDL3). The plant receptor-like protein kinases with leucine-rich repeat motif (LRR-RLK) is one of largest plant protein families and they are implicated in hormone and stress response pathways and in plant developmental processes [69]. Some members of LRR-RLK family and GDPL proteins participate in the formation of plant cell wall that acts as a barrier for both biotic and abiotic stresses [86,87]. In this work, we have detected about 2-fold reduction of *RLP1* and *GDPDL3* expression (−2.11-fold, −2.52-fold, respectively), which would account for the implication of this new tomato miRNA and its targets in the response to *P. syringae* infection.

## 5. Conclusions

In this work, miRNAs associated with stress response in tomato were identified and analyzed by high-throughput sequencing. We have detected 37 miRNA families in tomato plant leaves undergoing six different stress conditions, and five miRNAs were found differentially expressed under drought, heat (5 miRNAs), CPB damage (6 miRNAs), *P. syringae* infection (17 miRNAs), *B. cinerea* infection (3 miRNAs), and Hx acid treatment (12 miRNAs). Collectively, our results highlight the interest in knowing the miRNAs involved in the regulation of different plant stress responses, as well as their putative shared or related targets to explain how plants integrate molecular and physiological adaptation to stress. 

## Figures and Tables

**Table 1:** *S. lycopersicum* sRNA sequencing datasets. Statistics of sRNA sequences for six stress conditions.

Library	Replicates	Raw Reads	Reads in Analysis	Unique Reads in Analysis	Genome Mapped Reads	Unique Reads Mapped to Genome	Match Known miRNAS
CPB	CPB_control_1	2017580	1310283	202324	1007231	154699	87227
	CPB_control_2	2094577	1375345	213714	1073497	165203	108260
	CPB_control_3	1207208	781673	127111	598432	96578	88872
	CPB_1	1287488	892095	131365	678481	99049	70026
	CPB_2	1778426	1252398	178292	969344	136223	83870
	CPB_3	2165134	1479772	214253	1124057	163047	102550
*P. syringae*	Ps_control_1	2160524	1362621	221596	1027304	166724	220908
	Ps_control_2	1588889	922728	164896	667568	123168	171207
	Ps_control_3	1787979	1051329	173251	783434	129463	191246
	Ps_1	974244	549162	93482	405265	67620	253642
	Ps_2	1114462	590372	104438	441541	74500	291917
	Ps_3	464118	233552	42425	177202	29989	217635
*B. cinerea*	Bc_control_1	1663364	1228666	148546	931181	109353	71292
	Bc_control_2	1764492	1224461	169339	949056	125720	98452
	Bc_control_3	2321326	1651189	218069	1265836	163405	89453
	Bc_1	2452194	1789008	212957	1326215	154640	132166
	Bc_2	11464935	9559569	760151	7069617	537263	89613
	Bc_3	782598	556187	75621	388166	53376	67491
Drought	Drought_control_1	2686918	1922203	238355	1465597	181605	62462
	Drought_control_2	2980066	2139770	272662	1630658	205056	98298
	Drought_control_3	2865419	2197332	245822	1726340	190059	52195
	Drought_1	2827918	2059550	259159	1571717	198434	85571
	Drought_2	2720161	2081553	259423	1535046	198495	84087
	Drought_3	2950568	2118453	298178	1531771	224812	117450
Heat	Heat_control_1	1801710	1048696	191165	775561	143566	145078
	Heat_control_2	3224186	2180523	310215	1667525	234701	107871
	Heat_control_3	1969244	1183052	206622	895057	156249	197825
	Heat_1	2719328	1900640	268834	1375793	201450	104117
	Heat_2	2578226	1839653	304566	1412749	234794	86406
	Heat_3	1653823	1157590	167601	915105	128069	43457
Hexanoic acid	Hx_control_1	3589204	2863643	316033	2223853	238842	60636
	Hx_control_2	2633355	2006288	227564	1569173	171024	82970
	Hx_control_3	1334918	918326	145154	714753	109633	101580
	Hx_1	2284032	1643993	226204	1228535	166947	144444
	Hx_2	3098489	2407326	276169	1852663	210496	64147
	Hx_3	2363533	1830552	235801	1387512	177399	79684

CPB: Colorado potato beetle; *P. syringae*: *Pseudomonas syringae*; *B. cinerea*: *Botrytis cinerea*.

**Table 2:** Number of known miRNAs detected in *S. lycopersicum* plants under six different stress conditions. Only miRNAs present at least in two of the three libraries of each stress condition were considered.

Library	Known miRNAs
Control	100
CPB	65
*P. syringae*	86
*B. cinerea*	84
Drought	88
Heat	87
Hexanoic acid	29

**Table 3:** Number of novel miRNAs detected in *S. lycopersicum* plants under six different stress conditions. Only miRNAs present at least in two of the three libraries of each stress condition were considered.

Library	Novel miRNAs
Control	23
CPB	13
*P. syringae*	30
*B. cinerea*	21
Drought	19
Heat	25
Hexanoic acid	28

**Table 4:** Main features of eight representative novel *S. lycopersicum* miRNAs.

Name	Sequence	Length	% GC	Sly Chrom	ChromStart	ChromEnd	Strand	Minimum Free Energy (kcal/mol)
sly-newmiR3-5p	UAACUUCGUCUAGCUCGCCUUC	22	50.0	10	1709804	1709946	+	−57.1
sly-newmiR21-3p	ACCGCAGAAGCAUCAAUGUCC	21	52.4	3	10204269	10204358	+	−56.1
sly-newmiR22-3p	GUUUGCAUAUGUCAGGAGCUUU	22	40.9	3	61786104	61786202	+	−37.0
sly-newmiR26-3p	GCGGUACCAAAUCGAGGCAA	20	55.0	4	30503172	30503253	−	−21.4
sly-newmir35-5p	GUGCCGUGAUGAUUAAUGCAUAA	23	39.1	6	37102293	37102390	+	−20.9
sly-newmiR36-3p	GGGAGAAGGGGUGCCUCCUCA	21	66.7	7	56792405	56792486	+	−26.5
sly-newmiR37-5p	CUGCCGAAGCUGUGGGAUGU	20	60.0	8	44161949	44162021	+	−19.1
sly-newmiR51-3p	GGUGGAGCAUGUGGUUUAAUUCG	23	50.0	2	33096229	33096295	−	−15.9

**Table 5:** Number of miRNAs differentially expressed in *S. lycopersicum* plants under six different stress conditions.

Library	Novel miRNAs	Known miRNAs	Total miRNAs	Upregulated miRNAs	Downregulated miRNAs	Stress Specific miRNAs
CPB	4	2	6	4	2	4
*P. syringae*	9	8	17	10	7	14
*B. cinerea*	1	2	3	3	0	2
Drought	4	1	5	3	2	3
Heat	3	2	5	4	1	4
Hexanoic acid	12	0	12	7	5	8

**Table 6:** Number of predicted targets for known and novel differentially expressed miRNA in *S. lycopersicum* plants under six different stress conditions.

Library	Known miRNAs Targets	Novel miRNAs Targets	Total miRNAs Targets	Stress Specific Targets
CPB	6	19	25	16
*P. syringae*	50	55	105	69
*B. cinerea*	4	3	7	6
Drought	18	23	41	25
Heat	10	3	13	11
Hexanoic acid	0	26	26	16

**Table 7:** Target prediction for four selected miRNAs. Predicted at psRNA target web server (http://plantgrn.noble.org/v1_psRNATarget/) and miRNAconsTarget tool from sRNAtoolbox. Expectation (complementarity between small RNA and their target transcript, lower is best, from 0 to 5), UPE (maximum energy to unpair the target site, lower is best), multiplicity (number of putatives sites in the target).

miRNA	Target	Expectation	UPE	miRNA Start	miRNA End	Target Start	Target End	miRNA Aligned_Fragment	Target Aligned Fragment	Inhibition	Target Description	Multiplicity
*sly-miR167c-3p*	Solyc02g086840.2	3.5	15.25	1	20	740	759	GGUCAUGCUCGGACAGCCUC	GGGGCUUUCUGAGCAUGAUA	Cleavage	Kinesin light chain-like protein	1
*sly-miR172a*	Solyc01g108180.2.1	4.5	22.47	1	20	2010	2029	AGAAUCUUGAUGAUGCUGCA	CUCAGGAUCAUCAAGAGUCU	Cleavage	Pentatricopeptide repeat-containing protein (PPR)	1
*sly-miR171a*	Solyc08g078800.1.1	0.5	18.53	1	21	396	416	UGAUUGAGCCGUGCCAAUAUC	GAUAUUGGCGCGGCUCAAUCA	Cleavage	GRAS family transcription factor (GRAS40)	1
	Solyc08g081890.2.1	2.5	17.79	1	20	3647	3666	UGAUUGAGCCGUGCCAAUAU	AUAUUGGCAUGGCUCUAUCG	Cleavage	Multidrug resistance protein ABC transporter family	1
*sly-newmiR22-3p*	Solyc12g014570.1.1	3.0	17.57	1	22	1419	1440	GUUUGCAUAUGUCAGGAGCUUU	GGAAUUCUUGACAUAUGCAAAA	Cleavage	Glycerophosphoryl diester phosphodiesterase family protein (GDP)	1
	Solyc01g101100.2.1	3.0	16.50	1	20	1270	1288	GUUUGCAUAUGUCAGGAGCU	GGCUCCUGA-AUAUGCAAAU	Translation	Receptor-like protein kinase (RLP1)	1

## Supplementary Materials

The following are available online at https://www.mdpi.com/2073-4425/10/6/475/s1, Table S1: RT-qPCR primers for miRNAs and targets. Additional file 2: Table S2. Top miRNAs for each stress condition. Additional file 3: Table S3. Conserved miRNAs for each stress condition with sequence and reads. Additional file 4: Table S4. Novel miRNAs identified with characteristics. Table S5: Expression analysis data of high-throughput sequencing. Additional file 6: Table S6. Expression analysis data of high-throughput sequencing and RT-qPCR of ten selected stress-responsive miRNAs. Additional file 7: Table S7: Target prediction for conserved and novel differentially expressed miRNAs. Additional file 8: Table S8: GO terms for targets of conserved and novel differentially expressed miRNAs. Additional file 9: Table S9: Pairwise correlation values for all libraries.
